# Multiparity and Spontaneous Coronary Artery Dissection in the Postpartum Period

**DOI:** 10.1155/2012/420629

**Published:** 2012-12-25

**Authors:** Müntecep Aşker, Selvi Aşker, Özgür Gürsu

**Affiliations:** ^1^Department of Cardiology, Van Higher Education Training and Research Hospital, Van 65100, Turkey; ^2^Department of Chest Disease, Van Higher Education Training and Research Hospital, Van 65100, Turkey; ^3^Department of Cardiovascular Surgery, Van Higher Education Training and Research Hospital, Van 65100, Turkey

## Abstract

Spontaneous coronary artery dissection (SCAD) is a deadly cause of myocardial infarction (MI) that mainly affects otherwise healthy, young females. Forty percent of patients die suddenly or within a few hours of symptom onset. We examine the case of a young female who presented with chest pain. She developed ST elevations in anterolateral leads mimicking ST elevation MI. Cardiac catheterization was done and showed a middle left anterior descending (LAD) dissection. The patient underwent primary percutaneous transluminal coronary angioplasty with coronary stent placed in the LAD.

## 1. Introduction

Postpartum spontaneous coronary artery dissection (SCAD) is a rare cause of acute myocardial infarction. The incidence of acute myocardial infarction during pregnancy and puerperium reported by various studies ranges from 2.8 to 10 cases per 100,000 deliveries [1–3]. Survivors of the initial event generally have a good long-term prognosis, and around 70% of cases are diagnosed following the patient's death [4]. Approximately, one in four female patients with spontaneous coronary artery dissection is in the peripartum period, most commonly in the third trimester of pregnancy or in the early postpartum (usually within the two weeks) period. The indicated therapeutic approach to these patients is not well defined because of the small number of cases. Stenting is the considered therapy in case of a well-localised dissected lesion in a single vessel not involving the left main stem. To our knowledge, the case is unique as it presents with the youngest grand multiparous woman (28 years, gravida 10, para 8) with postpartum spontaneous coronary artery dissection reported in the literature.

## 2. Case Report

A 28-year-old woman gave birth to a healthy male infant following a normal, uneventful vaginal delivery (gravida 10, para 8). The patient was admitted to the emergency department six days postpartum with the acute onset, two hours prior, of substernal chest pain radiating to the left arm and interscapular region. The patient had no coronary risk factors such as hypertension, diabetes, or dyslipidemia, had no family history of heart disease, and did not have a history of oral contraceptive use. She did not smoke and did not use alcohol or drugs.

On physical examination, her blood pressure was 110/65 mmHg and her heart rate 89 beats/min, temperature of 37.2°C, and oxygen saturation of 94%. Auscultation of heart and lung fields was normal. Physical examination revealed no signs of heart failure. Chest X-ray was normal. Electrocardiography demonstrated the presence of ST-segment elevation in leads I, aVL (both 1 mm) and V1–V5 (all 3 mm), along with reciprocal ST segment depression in leads II, III, and aVF (all 2 mm). The initial laboratory analyses were within normal limits.

**Figure 1:**
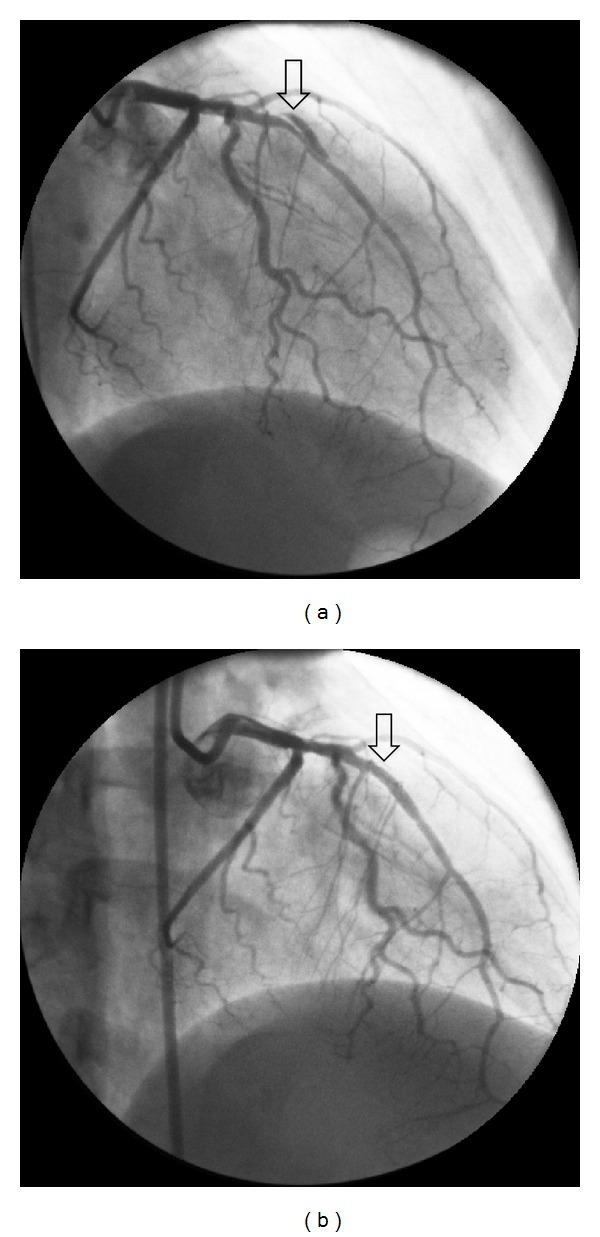
The left anterior descending artery showed spontaneous dissection (arrow). There was a 80% stenosis of the true lumen of the mid-LAD caused by external compression by the hematoma in the false lumen (Figure 1(a)). Bare-metal stent (3.0 × 18 mm) (arrow) showed in the left anterior descending artery (Figure 1(b)).

Subsequent cardiac catheterization revealed spontaneous dissection of the left anterior descending artery (LAD) (Figure 1(a)). There was a 80% stenosis of the true lumen of the mid-LAD caused by external compression by the hematoma in the false lumen. The right coronary artery (RCA) and left circumflex artery (LCX) were angiographically normal. The patient underwent percutaneous transluminal coronary angioplasty with bare-metal stent (3.0 × 18 mm) placed in the LAD (Figure 1(b)). We continued with low-molecular-weight heparin, clopidogrel, ramipril, metoprolol, and aspirin. The patient was discharged with good hemodynamic balance on the fourth day after admittance. One month after the control examination, the patient had no chest pain at all. Follow-up echocardiography revealed normal left ventricular function.

## 3. Discussion

The etiology of cardiac events in otherwise healthy young women is unclear. Independent predisposing factors that have been associated with an increased risk of infarction in pregnant women are age over 30 years, the third trimester of pregnancy, multiparity, hypertension, eclampsia and preeclampsia, diabetes, smoking, thrombophilia, need for blood transfusion, and appearance of infection following delivery [1, 2, 4]. Multiparity seems to be a risk factor for this patient.

There are more than 60 cases of postpartum SCAD in the literature and, in most cases, SCAD was proved on angiography to be an intimal flap or a spiral dissection [4]. The main vessel affected (78% of patients) was the anterior descending branch, followed by the circumflex artery (29%) and the right coronary artery (26%). It is noteworthy that in 24% of patients the dissection included the main stem of the left coronary artery, while in 40% of patients there were dissections in more than one vessel [4]. The present case had isolated dissection of the LAD. 

The indicated therapeutic approach to these patients is not well defined because of the small number of cases. Therapy should depend on the persistence of myocardial ischaemia, the area at risk, and the number of vessels involved. Medical therapy is generally advocated for nonischemic cases in which coronary blood flow is uncompromised. Heparin, beta blockers, diuretics, nitrates, and aspirin can be used with appropriate hemodynamic monitoring [5]. Thrombolytic therapy may be useful in arteries where the intramural haematoma is compressing the true lumen, allowing the latter to reexpand [6]. Stenting is the considered therapy in case of a well-localised dissected lesion in a single vessel not involving the left main stem [7]. In case of multivessel or left main stem involvement, surgical revascularisation seems the most controlled strategy. In patients with ongoing myocardial ischaemia or in patients refractory to medical treatment, bypass surgery also is the most appropriate therapy [8–10]. The present case had ST elevation in ECG. We have demonstrated isolated dissection of the LAD on coronary angiography and successful treatment with stent implantation. 

## 4. Conclusions

To our knowledge, the case is unique as it presents with youngest grand multiparous woman with postpartum SCAD reported in the literature. Immediate coronary angiography is essential to establish an early diagnosis. Medical treatment and percutaneous coronary intervention are usually sufficient to restore coronary flow and stable hemodynamics.
